# Prevalence and risk factors of gallbladder polyps in primary health care centers among patients examined by abdominal ultrasonography in Qatar: a case–control study

**DOI:** 10.5339/qmj.2021.48

**Published:** 2021-10-07

**Authors:** Tamer A. Ali, Abdelwahed Samir Abougazia, Ahmed Sameer Alnuaimi, Mona A. M. Mohammed

**Affiliations:** ^1^Radiology Department, PHCC, Doha, Qatar E-mail: dr.tamer1973@gmail.com; ^2^Research department, PHCC, Doha, Qatar; ^3^RadiologyDepartment, MC, MoPH, Doha, Qatar

**Keywords:** Gallbladder, polyp, prevalence, risk factor, stone, ultrasound

## Abstract

Background: Gallbladder (GB) polyps are raised lesions from the GB wall and projected into its lumen. The prevalence of GB polyps ranged between 4.3% and 12.3%. The clinical presentation of GB polypoid lesions vary, can be nonspecific and vague, and may be asymptomatic. Identifying malignant and premalignant polyps is important to provide treatment early and prevent cancer spread or development of malignancy. Ultrasonography (US) is the first imaging modality widely used in abdominal imaging. It is a noninvasive, rapid, painless, and safe imaging technique, with no radiation; thus, it is considered the best available examination with good sensitivity and specificity for GB polyps.

Aim of the work: This study aimed to determine the relative frequency of the GB polyps and its risk factors among patients who underwent abdominal US in Primary Health Care Corporation, Qatar.

Materials and methods: This was quantitative multicenter observational case–control study nested in a cross-sectional design. For the cross-sectional top-level study, the first step was to assess available abdominal ultrasound studies for the presence of GB polyps and stones. The second step was to perform a case–control study with three groups (a case group and two control groups; first, participants without GB stones and GB polyps; second, patients with GB stones but without GB polyps).

Results: The study evaluated the GB images of 7156 individuals. The overall prevalence of GB polyps was 7.4% in the study population. Specifically, the overall prevalence of solitary GB polyp was 4.2% and that of multiple GB polyps was 3.2%. Regarding the size distribution of GB polyps in positive cases, 89.4% were < 6 mm, 9.3% were 69 mm, and 1.3% were ≥ 10 mm. Prevalence rate of selected comorbidities were as follows: liver disease, 1.8%; diabetes mellitus, 25.5%; hypertension, 25.5%; and dyslipidemia, 29.8%. The prevalence in male and female patients was 7.7% and 7%, respectively. The prevalence of GB polyps was higher in south-eastern patients (21.4% of positive cases) and was the highest in the overweight group (8.8%). A higher prevalence was noted in the hypertensive group (hypertensive group, 9.8%; non-hypertensive group, 6.6%) and dyslipidemia group (dyslipidemia group, 7.8%; no dyslipidemia group, 7.2%). Moreover, a higher prevalence was noted in hepatitis B surface (HBS)-positive group (15%) than in the HBS-negative group (8.2%) and slightly higher in *Helicobacter pylori* antigen positive group than in the negative group.

Conclusion: Abdominal US is an important and commonly used imaging modality in the detection of GB polyps. In this study, the prevalence of GB polyps was approximately 7.4%, with higher prevalence in participants who were overweight and had diabetes mellitus, hypertension, and dyslipidemia.

## Background

Gallbladder (GB) polyps are raised lesions from the GB wall and projected into its lumen. Most of them are asymptomatic and incidentally discovered during abdominal ultrasonography (US) performed for other reasons. GB polyps can be classified into benign and malignant polyps (carcinoma). While a great proportion of GB polyps are benign, a big concern is always regarding the presence of malignant change because most patients have late presentation, diagnosed post-cholecystectomy, and have late stage disease and poor prognosis.^1-6^

In literature, the prevalence of GB polyps varies, but it ranges between 4.3% and 12.3% in many studies.^7-11^ In a large Japanese study, GB polyps were reported in 5.6% with a male predominance.^9,10^ In Chinese patients, GB polyps were identified in 6.7% of the patients.^8,9^ Other studies have reported GB polyps in 4.6% of male and 4.3% of female individuals in Denmark,^11^ 1.5% in Germany,^12^ and 0.32% in India.^13^

Many studies have investigated the risk factors for GB polyps. Demographic variations were observed, such as a high prevalence of GB polyps in the third to fifth decades of life.^5,9,11^ Some studies have shown a higher prevalence of GB polyps in men than in women,^5,9,12,14^ while no gender predominance was detected in other studies.^8,11^ Several earlier studies have identified some risk factors for GB polyps, including obesity, diabetes mellitus (DM), chronic hepatitis, serum cholesterol level, and metabolic syndrome.^15–19^

The clinical presentation of GB polypoid lesions vary, can be nonspecific and vague (such as nausea, vomiting, and occasional pain in the right hypochondrium), may be asymptomatic, and incidentally noted during abdominal US performed for other reasons.^3,19,20^ However, no significant difference was found in the presenting symptoms of benign and malignant polyps,^18^

The potential malignant transformation of GB polyps is a big concern; therefore, early detection and management of malignant ones are critical for treatment and long-term survival. Many studies have reported that GB polyps progress into cancer and adenomatous polyps containing carcinoma in situ.^21-24^ Adenomatous polyps appear to have the highest risk of malignant transformation. In a previous study of 300 randomly selected GBs at cholecystectomy, 19% of sessile adenomas showed small foci of moderate cellular atypia and 31% of them were positive for carcinoembryonic antigen.^23^ Another study of 1605 resected GBs also supported the adenoma–adenocarcinoma sequence.^21^

More than 178,000 new cases of GB cancers are diagnosed annually, making it the 20th most common cancer worldwide. The highest incidence of GB cancer is reported in South America and Asia, while the lowest is reported in North America and United Kingdom.^5^ Survival in GB cancer significantly varies. However, the 5-year survival rate can be as high as 80% in patients with in situ disease and can be at 8% and 2% in cases with lymph node involvement and stage 4b, respectively. These demonstrate the importance of identifying malignant and premalignant polyps to enable early treatment and thus prevent cancer spread or development of malignancy.^5,21-32^

Qatar is experiencing fast development with mega projects that led to high influxes of migrant workers and professionals from different countries, resulting in a multi-ethnic young adult population with continuous and high demographic turnover.^25^ This would reflect substantially on the epidemiology and clinicopathological characteristics of diseases prevalent in Qatar.^26^

US is the first imaging modality widely used in abdominal imaging. It is a noninvasive, rapid, painless, and safe imaging technique, with no radiation^27^; thus, it is considered the best readily available examination with good sensitivity and specificity to GB polyps. In some previous studies, the sensitivity of abdominal US in the diagnosis of GB polyps is higher than that of computed tomography (CT).^3,27,28,29^

The vision of the Primary Health Care Corporation (PHCC) is to be the leader in transforming the health and well-being of the people's lives in Qatar. At present, PHCC provides abdominal ultrasound service that can detect GB polyps with high sensitivity. However, to date, no data are available on the actual incidence and epidemiology of GB polyps in Qatar.^26^

## Aim Of The Work

This study aimed to determine the relative frequency of GB polyps and its association with several risk factors among patients who underwent abdominal US in PHCC.

## Materials And Methods

### Study settings

PHCC is a publicly funded primary care provider in Qatar. Majority of the country's population is registered with PHCC. It has 27 health centers across Qatar that uses an integrated electronic medical record (EMR) system. Every Qatar resident is eligible to register with a PHCC health center.

### Study design

This is a quantitative multicenter analytic cross-sectional study.

### Study population

The study population included all EMRs of PHCC-registered individuals (CERNER) who underwent an abdominal US in the PHCC Radiology Departments for any reason. A valid GB ultrasound image should be available on the official PHCC EMR system (RIS PACS system) during the 1-year study period from January 1, 2020, to December 31, 2020. Excluded from this group are individuals with a history of surgery on the GB or any biliary intervention.

### Study variables

The following variables were extracted from the electronic health record system for the targeted study population: The sociodemographic variables (age, gender, and nationality), personal history of biliary interventions, first available body mass index (BMI) measurement, presence of comorbidities (including type II DM, dyslipidemia, and arterial hypertension), smoking habit, past history of chronic liver disease, gastric *Helicobacter pylori* test, viral hepatitis, and viral hepatitis status (hepatitis surface antigen [HBS] and hepatitis C virus [HCV] antibodies). The list of comorbid conditions was identified in the EMR using preselected SNOMED codes.

### Data collection

The PHCC EMR system uses SNOMED codes, which are systematically organized computer processable collections of medical terms providing codes, terms, synonyms, and definitions used in clinical documentation and reporting. These codes are quality controlled and reviewed by the Business Health Intelligence (BHI) department of PHCC. BHI is responsible for translating SNOMED codes into International Classification of Disease the Tenth Revision codes and continuously updating the coding manual with any new code used in the organizational database at a monthly interval. BHI provided a full list of variables for the study population using filters created for the purpose of the study.

The study team examined the available abdominal ultrasound scans for the presence of GB polyps and stones. The polyps were identified by a combination of the following characteristics on US images: a fixed hyperechoic material protruding from the GB wall into the lumen with no acoustic shadow and does not shift with positional change.^30^ The number and diameter of the largest polypoid lesion were recorded.

### Data cleaning

Duplicates were removed. A total of 82 individuals had two sets of abdominal ultrasound scans performed during the study period. In these individuals, both sets of US images were evaluated to exclude inconsistencies between findings, and only one record per study participant was included. US images of 7645 participants were extracted from the records. Moreover, 489 of the remaining patients had no images of the GB that can be used for the assessment, and these images were also deleted from the database. The US images of the GB of the remaining 7156 individuals were evaluated.

### Data analysis

SPSS version 23 (IBM Corp., Armonk, NY, USA) was used for the statistical analysis of data. Data cleaning involved a range of checks (e.g., checking dates to be within the specified study time and checking for completeness of visual triage questionnaire). The frequencies of the selected variables were assessed first. The prevalence ratio (PR) was used to measure the strength of the association between two categorical variables. The significance of these associations was assessed using Chi-square test of independence. The level of significance was assumed at *p* < 0.05.

The 1-year prevalence (per 100 persons) of GB polyps was calculated using the following formula, and the same applies for the calculation of the prevalence of GB stones:


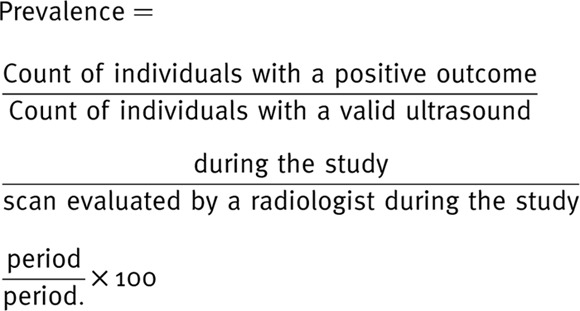


The PR was used to measure the strength of the association between a dichotomous independent variable (a specific group compared with a reference group) and a dichotomous outcome variable (positive for polyp). The PR is equal to the ratio between the prevalence of an outcome (GB polyps) among those with risk factors divided by the prevalence among those without the risk factor (comparison group). The logarithm method was used in the calculation of confidence intervals for the PR.

A multiple logistic regression model with selected factors as independent variables and depression as the dependent variable was used. The model assessed the risk of having the outcome for each explanatory variable after adjusting for the effect of other confounders included in the model. The model provides the following parameters:

• P value for the model: To generalize the results obtained, the model should be significant.
• Predictive power of the model: The overall predictive power was expressed as a percentage of the study participants being classified correctly based on the calculated parameters.
• Adjusted odds ratio (OR)): This was defined as the risk of having the outcome in the presence of a specific risk factor. Each OR was adjusted for the effect of other explanatory variables included in the model to represent a net effect of each factor on the risk of having the outcome. The OR for different explanatory variables in a participant is additive; in other words, the risk of having the outcome in a specific participant is the sum of the OR for the whole set of explanatory variables.
• P value for OR: This reflects the significance of the calculated OR.^31^

### Quality control measures

In the preparation phase of the study, an extensive review of literature was undertaken. The authors were responsible for the data collection in collaboration with the BHI.

### Ethical considerations

The study presented a minimal risk of harm to its participants, and the data collected were anonymized by the BHI. Overall, the study was conducted with integrity according to generally accepted ethical principles. The research proposal was approved by PHCC's Research Sub-Committee (PHCC/DCR/2020/01/006).

## Results

**Figure 1.**
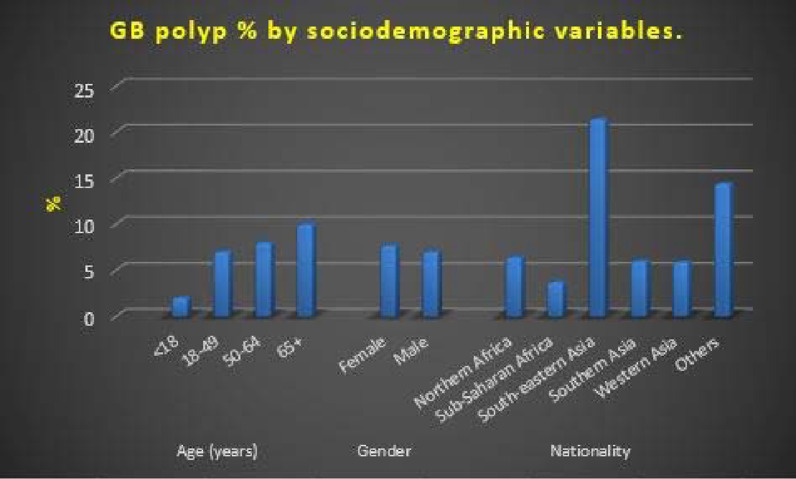
Frequency distribution of gallbladder polyp in the study sample by sociodemographic variables

**Figure 2.**
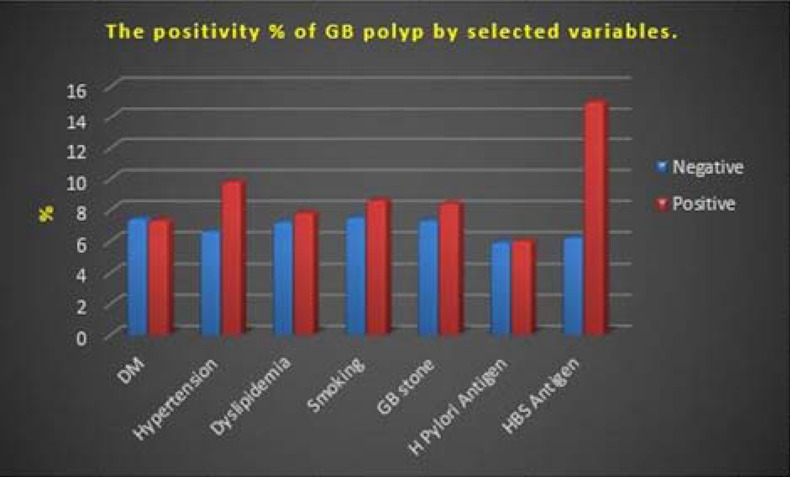
Prevalence of selected comorbidities in patients with polypoid lesions of the gallbladder

**Figure 3.**
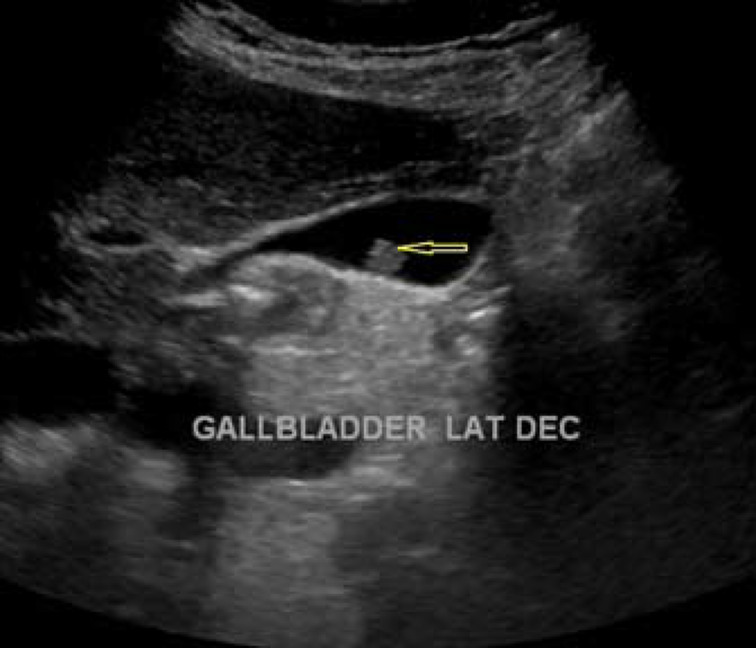
Ultrasound image showing a gallbladder polyp (yellow arrow)

**Figure 4.**
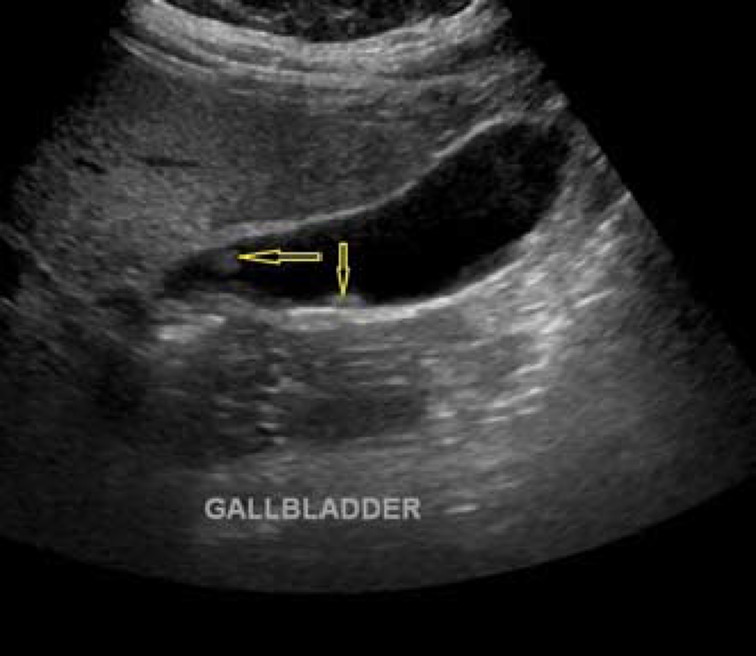
Ultrasound image showing multiple gallbladder polyps (yellow arrows)

**Figure 5.**
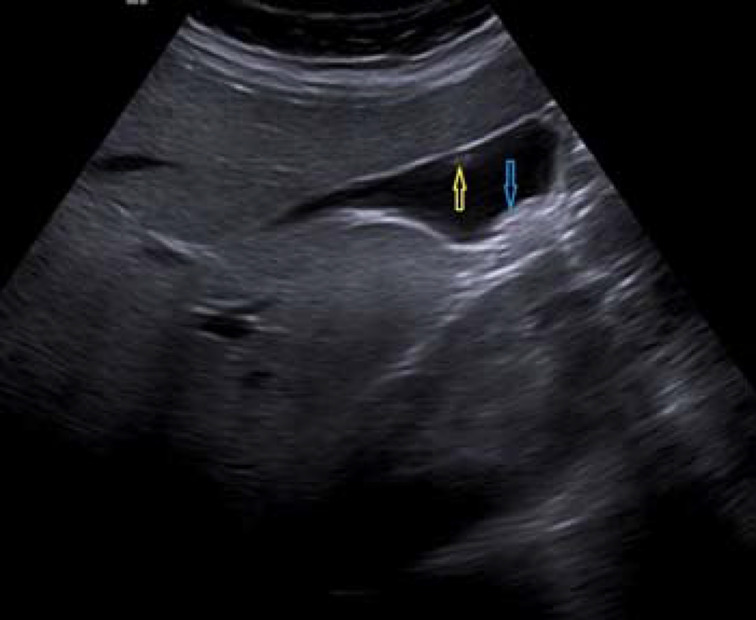
Ultrasound image showing gallbladder polyp (yellow arrow) and stones (blue arrow)

In total, GB US images of 7156 individuals were evaluated in this study (Figures 1–5). Female participants constituted 54.8% of the study population. Only 4.8% of the study sample was 65 years of age and older, while 5.6% were < 18 years old. The majority of the participants (70.2%) were young adults. More than a third of the recruited samples were overweight (35.8%), and another 44% were obese. In addition, those who ever smoked constituted 17.4% of the study population (Table 1).

PHCC service users are multinationals. Three of the nationality groups constituted 87.2% of the total sample: Western Asia, Southern Asia, and Northern Africa (35.8%, 28.6%, and 22.8% respectively) (Table 1).

The overall prevalence of GB polyps was 7.4% (95% confidence level in the reference population ranged from 6.8% to 8%). In addition, GB stones were detected in 9.7% of the recruited sample (the 95% confidence level in the reference population ranged from 9% to 10.4%) (Tables 2 and 3).

Solitary and multiple GB polyps were found in 4.2% and 3.2% of the study participants, respectively. Regarding the size, 89.4% of the GB polyps (considering the largest in case of multiple polyps) were < 6 mm, while only 1.3% of the polyps were >10 mm (Table 2).

As shown in Table 4, the prevalence of selected comorbidities was 1.8% for chronic liver disease, 25.5% for DM, 25.5% for hypertension, and 29.8% for dyslipidemia. In addition, the positivity rates of selected test results were as follows: *H. pylori* antigen (7.9%), HBS antigen (0.3%), and HCV antibodies (0.8%) (Table 5).

The risk of having GB polyps was assessed for selected independent variables (Table 6). The risk of having GB polyps is significantly increased with advanced age, with 4.5 times higher risk in those aged 65+ years than in those aged < 18 years. All nationality groups were associated with an increment in risk when compared with the Sub-Saharan Africans, which had the lowest prevalence rate of GB polyps (3.7%). Southern Asians were significantly associated with the highest increase in risk of 5.8 times when compared with Sub-Saharan Africans. Among the remaining explanatory variables, only hypertension was associated with a significant increase in the risk of having GB polyps by 48%. In addition, testing positive for HBS antigens was associated with an obvious (but insignificant) increase in risk of GB by 2.4 times. All other independent variables tested were not significantly or were significantly associated with an increase in GB polyp risk. These variables include, gender, BMI, liver disease, DM, dyslipidemia, GB stones, ever smoking, *H. pylori* antigen, and HCV antibodies.

A multiple logistic regression model was used to assess the net and independent effect of a set of explanatory variables on the risk of having GB polyps (Table 7). The model was significant and associated with an overall predictive accuracy of 92.2%. Only age, South-eastern Asia nationality, and hypertension were significantly associated with the risk of having GB polyps after adjusting for the possible confounding effects of other explanatory variables included in the model (gender, liver disease, DM, dyslipidemia, BMI categories, GB stones, and smoking status). Belonging to an older age group (65+ years) is associated with a significant increase in the risk of having GB polyps by 63%. Being a South-eastern Asian is associated with a significant increase in risk of having GB polyps by 3.7 times, when compared with the remaining nationality groups. Having hypertension significantly increased the risk of having GB polyps by 39% after controlling for other explanatory variables included in the model.

## Discussion

Abdominal US is an important and commonly used imaging modality in the detection of GB polyps. The incidence of GB polyps detected by abdominal US increased, which can be attributed to the increased and widespread use of abdominal US (main). Additionally, this increase in the incidence can be partially attributed to the higher prevalence of metabolic syndrome.^33-34^

This study included a total of 7156 individuals.

Providing the accurate prevalence of GB polyps is quite difficult, as most GB polyps are incidentally discovered in ultrasound studies. GB polyps were reported in 5.6% in a large Japanese study, while it was 6.7% in Chinese participants.^8,9^ More recent studies have shown a higher prevalence, i.e., 8.5%^35^ and 9.96%,^1^ and this can be attributed to the wider use of US in health checkups.^1^ In the present study, the overall prevalence of GB polyps was comparable with those in previous reports. In addition, GB stones were detected in 9.7% of the recruited sample.

The present study showed that the risk of having GB polyps significantly increased with advancing age, reaching 4.5 times higher risk in those aged 65+ years than in those aged < 18 years. In addition, differences in the relative frequency of GB polyps were found among nationality groups. Southern Asians were associated with the highest increase in risk of 5.8 times when compared with Sub-Saharan Africans.

Other studies have reported extreme variations in the prevalence of GB polyps among nationalities. A prevalence of 4.3%–4.6% was reported in Denmark,^11^ 1.5% in Germany,^12^ and 0.32% in India.^13^ In a study of Korean individuals who underwent health screening examinations, the estimated average prevalence of GB polyps was 2.94%.^1,36^

In the current study, the prevalence of GB polyps was slightly higher in female than in male (7.7 versus 7%, respectively) individuals. Similarly, small differences were reported between male and female individuals in Denmark^11^ (4.6% in male versus 4.3% for female individuals) and Korea^36^ (3.63% in male versus 2.09% in female individuals).

In the present study, the prevalence of the selected comorbidities was as follows: 1.8%, chronic liver disease; 25.5%, DM; 25.5%, hypertension; and 29.8%, dyslipidemia. In addition, the positivity rates of selected test results were as follows: *H. pylori* antigen (7.9%), HBS antigen (0.3%), and HCV antibodies (0.8%).

The most common GB polypoidal lesions are those containing cholesterol, and this was explained by some authors in that cholesterolosis is the result of the direct deposition of cholesterol from the blood,^14^ with cholesterol metabolism changes in the liver and impaired mucosal esterification of free sterols from the bile. Cholesterol polyps may be detached and causes GB-like clinical presentation such as biliary colic or obstruction.^18^

Based on this assumption, previous studies have investigated whether blood lipids are possible risk factors for GB polypoid lesions and concluded that blood cholesterol concentration is not an independent risk factor.^5,7,8,12,16^ In the present study, patients with dyslipidemia had slightly higher incidence for GB polyps (7.8 %) than patients without dyslipidemia (7.2%). This finding matches with earlier study^37^ that reported the association between dyslipidemias and GB polyp formations.

Among the remaining explanatory variables, only hypertension was associated with a significant increase in the risk of having GB polyps by 48%. In addition, testing positive for HBS antigen was associated with a remarkable (but insignificant) increase in the risk of GB by 2.4 times. All the other tested independent variables had no significant or obvious association with an increase in GB polyp risk. These variables include gender, BMI, liver disease, DM, dyslipidemia, GB stones, smoking status, *H. pylori* antigen and HCV antibodies.

Some studies have shown the role of hyperglycemia in the formation of biliary stones by inhibiting GB contraction and liver secretion of bile^38–39^; however, some authors could not confirm whether hyperglycemia is a risk factor for GB polypoid lesions.^1^ In the present study, patients with DM did not have a higher prevalence of GB polyps than patients without DM.

While chronic hepatitis B (CHB) can cause abnormal GB changes such as altered GB volume and wall thickening, the association of CHB with GB polypoid lesions is still not clear. While some authors have suggested the direct effect of hepatitis B virus in bile on the GB mucosa, others suggested that hepatic parenchyma inflammatory changes can affect the GB mucosa.^1^ Some earlier studies have reported CHB as a risk factor for GB polypoid lesions.^1,40-41^ The results of the present study were in agreement with those of previous studies because CHB was found to be an independent risk factor for GB polypoid lesions. In the present study, a higher prevalence of GB polypoid lesions was noted in patients positive for HBS antigen (15%) than in those negative for it (6.2%)

Gastric *H. pylori* is known to be commonly associated with formations of GB stones^41,42^; a previous study^1^ also showed higher rate of *H. pylori* infection in patients with GB stones and polypoid lesions, which support the finding that *H. pylori* infection is related to stone formation rather than GB polypoid lesions. In the present study, no significant higher prevalence of GB polyps was identified in positive *H. pylori* cases (6%) when compared with *H. pylori-*negative cases (5.9%).

Small GB polyps are considered of lower malignant potential than larger polyps,^33^ and this was confirmed by Kubota et al.^43^ who showed that 2/7 of GB polyps < 5 mm found by cholecystectomy were adenomas.

In the present study, solitary GB polyp was found in 4.2% of the study participants, while multiple ones were found in 3.2% of the study participants. Regarding the size, majority (89.4%) of GB polyps (considering the largest in case of multiple polyps) were < 6 mm, only 1.3% of the polyps were >10 mm, and polyps of 6–9 mm were found in 9.3% in participants with GB polyps.

Compared with multiple polyps, a solitary GB polypoid lesion is assumed to be more liable for the malignant changes^42,44^; however, the size of the polyps may play a more important role in malignant changes.^1^ In the present study, solitary GB polyp was more common than multiple ones (4.2% versus 3.2%).

A multiple logistic regression model was used in the present study to assess the net and independent effects of a set of explanatory variables on the risk of having GB polyps. The model was significant and associated with an overall predictive accuracy of 92.2%. Only age, South-eastern Asian nationality, and hypertension showed a significant association with the risk of having GB polyps after adjusting for the possible confounding effects of other explanatory variables included in the model (gender, liver disease, DM, dyslipidemia, BMI categories, GB stones, and smoking status). Being in an older age group (65+ years) is associated with a significant increase in the risk of having GB polyps by 63%. Compared with the remaining nationality groups, being a South-eastern Asian is associated with a significant increase in the risk of GB polyps by 3.7 times. Having hypertension significantly increases the risk of having GB polyps by 39% after controlling for the other explanatory variables included in the model.

This study has some limitations. The study included individuals who were referred for US, and this may result in the difference when compared with the prevalence in the general population.

## Conclusion

Abdominal US are an important and commonly used imaging modality in the detection of GB polyps. In the present study, the prevalence of GB polyps was approximately 7.4%, with a higher prevalence in those with overweight, DM, hypertension, and dyslipidemia. Follow-up studies in high-risk groups are recommended for the early detection of any malignant changes.

### Competing interests

The authors declare that they have no competing interests.

### Ethical considerations

The study was approved by the Primary Health Care Corporation Research Committee and the Research Section in the Department of Clinical Affairs.

### Consent for publication

All authors read and approved the final manuscript.

### Availability of data and materials

The data that support the findings of this study are available on request from the corresponding author.

### Acknowledgment

We wish to acknowledge the Primary Health Care Corporation (Qatar) as a research funding agency for this article, and we wish to acknowledge the help provided by Research Department of the PHCC.

## Figures and Tables

**Table 1:** Frequency distribution of gallbladder polyp in the study sample by sociodemographic variables.

	N	%

**Age (years)**		

< 18	398	5.6

18–49	5023	70.2

50–64	1389	19.4

65+	346	4.8

Total	7156	100.0

**Gender**		

Female	3920	54.8

Male	3236	45.2

Total	7156	100.0

**BMI categories (kg/m^2^)-adults only**		

Abnormally low ( < 18.5)	59	1.1

Acceptable (18.5–24.9)	1056	18.8

Overweight (25–29.9)	2005	35.8

Obese I (30–34.9)	1514	27.0

Obese II (35–39.9)	635	11.3

Obese III (40+)	338	6.0

Total	5607	100.0

**Nationality groups**		

Northern Africa	1628	22.8

Sub-Saharan Africa	191	2.7

South-eastern Asia	537	7.5

Southern Asia	2049	28.6

Western Asia	2564	35.8

Miscellaneous others	187	2.6

Total	7156	100.0

**Ever smoked cigarettes**		

Negative	4615	82.6

Positive	973	17.4

Total	5588	100.0


**Table 2:** Description of gallbladder polyps in the present study.

	n	%	95% confidence interval for the prevalence rate

**Gallbladder polyps**		

None	6627	92.6	(92 to 93.2)

Single	300	4.2	(3.7 to 4.7)

Multiple	229	3.2	(2.8 to 3.6)

Total	7156	100.0	

**Size of the largest gallbladder polyp**			

< 6 mm	473	89.4	(86.6 to 91.8)

6–9 mm	49	9.3	(7 to 12)

≥ 10 mm	7	1.3	(0.6 to 2.6)

Total	529	100.0	


**Table 3:** Prevalence of positive gallbladder findings.

Gallbladder findings (n=7156)	n	%	95% confidence interval for prevalence rate

Gallbladder Polyp	529	7.4	(6.8 to 8)

Gallbladder stones	693	9.7	(9 to 10.4)


**Table 4:** Prevalence of selected comorbidities in patients with polypoid lesions of the gallbladder.

(n=7156)	N	%

Comorbid conditions		

Liver disease	128	1.8

Diabetes mellitus	1826	25.5

Hypertension	1824	25.5

Dyslipidemia	2131	29.8


**Table 5:** Positivity rates of selected test results in patients with polypoid lesions of the gallbladder.

(n=7156)	N	%

Positive test results		

*H. pylori* antigen	562	7.9

Hepatitis B surface antigen	20	0.3

Hepatitis C antibodies	55	0.8


**Table 6:** Prevalence ratio for having gallbladder polyp by selected explanatory variables.

	Gallbladder polyps								

	Negative		Positive		Total		95% CI for		

	N	%	N	%	N	%	PR	PR	P

Age (years)									

< 18	389	97.7	9	2.3	398	100	Ref		

18–49	4648	92.5	375	7.5	5023	100	3.26	(1.7–6.26)	0.002

50–64	1280	92.2	109	7.8	1389	100	3.39	(1.73–6.63)	0.001

65+	310	89.6	36	10.4	346	100	4.52	(2.21–9.25)	< 0.001

Nationality groups									

Sub-Saharan Africa	184	96.3	7	3.7	191	100	Ref		

Western Asia	2412	94.1	152	5.9	2564	100	1.59	(0.76–3.34)	0.64[NS]

Southern Asia	1926	94	123	6	2049	100	1.62	(0.77–3.42)	0.63[NS]

Northern Africa	1523	93.6	105	6.4	1628	100	1.73	(0.82–3.66)	0.51[NS]

South-eastern Asia	422	78.6	115	21.4	537	100	5.78	(2.74 − 12.17)	< 0.001

Miscellaneous others	160	85.6	27	14.4	187	100	3.89	(1.74–8.71)	0.004

Gender									

Male	3008	93	228	7	3236	100	Ref		

Female	3619	92.3	301	7.7	3920	100	1.1	(0.93–1.3)	0.79[NS]

BMI categories (kg/m^2^)									

Acceptable ( < 25)	1022	91.7	93	8.3	1115	100	Ref		

Overweight (25–29.9)	1829	91.2	176	8.8	2005	100	1.06	(0.83–1.35)	0.98[NS]

Obese (30+)	2325	93.5	162	6.5	2487	100	0.78	(0.61–1)	0.27[NS]

Liver disease									

Negative	6503	92.5	525	7.5	7028	100	Ref		

Positive	124	96.9	4	3.1	128	100	0.41	(0.16–1.08)	0.33[NS]

Diabetes mellitus									

Negative	4934	92.6	396	7.4	5330	100	Ref		

Positive	1693	92.7	133	7.3	1826	100	0.99	(0.82–1.2)	1[NS]

Hypertension									

Negative	4982	93.4	350	6.6	5332	100	Ref		

Positive	1645	90.2	179	9.8	1824	100	1.48	(1.25–1.76)	< 0.001

Dyslipidemia									

Negative	4663	92.8	362	7.2	5025	100	Ref		

Positive	1964	92.2	167	7.8	2131	100	1.08	(0.91–1.29)	0.83[NS]

Ever smoked cigarettes									

Negative	4269	92.5	346	7.5	4615	100	Ref		

Positive	889	91.4	84	8.6	973	100	1.15	(0.92–1.44)	0.69[NS]

Gallbladder stones									

Negative	5992	92.7	471	7.3	6463	100	Ref		

Positive	635	91.6	58	8.4	693	100	1.15	(0.89–1.49)	0.78[NS]

*H. pylori* antigen									

Negative	983	94.1	62	5.9	1045	100	Ref		

Positive	528	94	34	6	562	100	1.02	(0.68–1.53)	1[NS]

Hepatitis B surface antigen									

Negative	1435	93.8	95	6.2	1530	100	Ref		

Positive	17	85	3	15	20	100	2.42	(0.84–6.99)	0.46[NS]

Hepatitis C antibodies									

Negative	1397	93.9	91	6.1	1488	100	Ref		

Positive	53	96.4	2	3.6	55	100	0.59	(0.15–2.33)	0.9[NS]


**Table 7:** Multiple logistic regression model with the risk of having GB polyps as the dependent (response) variable and selected explanatory variables.

	Partial OR	95% confidence interval	P

Age (years)			0.11[NS]

50–64 years compared with 18–49 years	1.06	(0.79 to 1.43)	0.68[NS]

65+ years compared with 18–49 years	1.63	(1.03 to 2.59)	0.038

South-eastern Asia nationality compared with the remaining nationality groups	3.76	(2.72 to 5.19)	< 0.001

Males compared with females	1.00	(0.78 to 1.3)	0.98[NS]

Liver disease	0.37	(0.12 to 1.18)	0.09[NS]

Diabetes mellitus	0.83	(0.62 to 1.1)	0.2[NS]

Hypertension	1.39	(1.05 to 1.83)	0.021

Dyslipidemia	0.91	(0.69 to 1.19)	0.47[NS]

BMI categories (kg/m^2^)			0.19[NS]

Overweight (25–29.9) compared with acceptable ( < 25)	1.13	(0.84 to 1.53)	0.41[NS]

Obese (30+) compared with acceptable ( < 25)	0.90	(0.66 to 1.23)	0.51[NS]

Having gallbladder stones	0.95	(0.67 to 1.35)	0.78[NS]

Ever smoked cigarettes	1.30	(0.96 to 1.75)	0.09[NS]

Constant	0.07	(0 to 0)	< 0.001


P (Model) < 0.001; Overall predictive accuracy = 92.2%
